# Network resilience of mutualistic ecosystems and environmental changes: an empirical study

**DOI:** 10.1098/rsos.180706

**Published:** 2018-09-12

**Authors:** Ellie Nagaishi, Kazuhiro Takemoto

**Affiliations:** Department of Bioscience and Bioinformatics, Kyushu Institute of Technology, Iizuka, Fukuoka 820-8502, Japan

**Keywords:** network science, network resilience, mutualistic networks, human impact, climate change

## Abstract

It is theorized that a mutualistic ecosystem's resilience against perturbations (e.g. species extinction) is determined by a single macroscopic parameter (network resilience), calculable from the network. Given that such perturbations occur owing to environmental changes (e.g. climate change and human impact), it has been predicted that mutualistic ecosystems that exist despite extensive environmental changes exhibit higher network resilience; however, such a prediction has not been confirmed using real-world data. Thus, in this study, the effects of climate change velocity and human activities on mutualistic network resilience were investigated. A global dataset of plant–animal mutualistic networks was used, and spatial analysis was performed to examine the effects. Moreover, the potential confounding effects of network size, current climate and altitude were statistically controlled. It was demonstrated that mutualistic network resilience was globally influenced by warming velocity and human impact, in addition to current climate. Specifically, pollination network resilience increased in response to human impact, and seed-dispersal network resilience increased with warming velocity. The effect of environmental changes on network resilience for plants was remarkable. The results confirmed the prediction obtained based on the theory and imply that real-world mutualistic networks have a structure that increases ecosystem resilience against environmental changes. These findings will enhance the understanding of ecosystem resilience.

## 1. Introduction

Understanding the dynamics of ecosystems is a significant challenge in ecology [1–4]. Specifically, the resilience of ecosystems against environmental perturbations (e.g. climate change) has attracted attention [5–7] in the context of biodiversity maintenance and environmental assessment [3,8]. According to [6], resilience is defined as a system's ability to adjust its activity so that basic functionality may be retained when errors, failures and environmental changes occur; it is a striking feature in real-world complex systems such as ecosystems. Ecological resilience has long been discussed theoretically [9,10] and is often considered to be related to the probability of species (co)extinction. Species coextinction can be considered a series of complex extinction cascades and is often explained in the context of stochastic processes [11]. For decades, the resilience of ecological assemblages has been theoretically studied using a network approach [12]. Networks describe the relationships among elements and are thus simple and powerful tools for describing complicated systems such as ecosystems. Ecological communities consist of a number of species that are connected via interspecific interactions, such as trophic and mutualistic relationships, and they are represented as networks (so-called *ecological networks*, in which nodes and edges correspond to species and interspecific interactions, respectively). As the availability of ecological data has increased, network science has also been applied to ecology [13], in addition to biology [14] and medicine [15]. Network science enhances the understanding of ecological resilience. For example, Allesina & Pascual [16] demonstrated that the consequence of species extinction resulting from the loss of a single species is predictable using eigenvector measures such as Google PageRank, under the assumption that a species is important if other important species rely on it for their survival. Vieira & Almeida-Neto [11] proposed a simple stochastic model for complex species coextinctions in mutualistic networks (e.g. pollination networks and seed-dispersal networks), and they showed that ecological resilience decreases with the level of connectedness (connectance or graph density). Fricke *et al*. [17] extended the stochastic model and found that seed-dispersal networks have an optimal structure that minimizes species coextinction. Using the stochastic model, Schleuning *et al*. [18] showed that mutualistic networks are more sensitive to plant than to animal extinction.

The studies reviewed above are primarily based on numerical simulation, but the recent theoretical study [6] on universal resilience patterns of complex networks is of particular interest because it provides an analytical framework of resilience for multi-dimensional systems. This study considered a mathematical model for mutualistic ecosystems and showed that a multi-dimensional complex system is reducible to an effective one-dimensional system. In particular, the study [6] postulates that a single resilience parameter (*β*_eff_), calculable from an ecological network, determines the state of the ecosystem (i.e. coexistence or extinction of species) against perturbations (e.g. species or interaction loss, interaction strength reduction, or any combination thereof). As the perturbations occur owing to environmental changes [19–22] (e.g. climate change and human impact), it has been hypothesized that mutualistic ecosystems that exist despite extensive environmental changes exhibit higher network resilience; however, such a hypothesis has not yet been evaluated in the real world.

In this context, macroecological approaches are useful. A significant amount of data on real-world ecological networks are available from such sources as GlobalWeb [8], the Interaction Web DataBase and the Web-of-Life Database, among others. Several studies have reported an association between environmental or external factors and ecological network structure, inspired by the hypothesis that ecological networks have an optimal structure that maximizes ecosystem stability against such perturbations [23–25]. For example, it was found that climate seasonality affects ecological networks [26]; in particular, the network structure of terrestrial ecosystems (pollination networks) was affected by temperature seasonality. Dalsgaard *et al*. [27] reported that the pollination network structure correlated with the historical rate of warming, and Sebastián-González *et al*. [28] demonstrated that the structure of seed-dispersal networks changed in response to human impact. In [29], it was reported that such a structural change in response to warming velocity and other forms of human impact was globally observed in various types of ecological networks (i.e. food webs, pollination networks and seed-dispersal networks). These studies indicated that the change in network structure was due to the perturbations (e.g. species/interaction loss) that occur subsequent to environmental changes; thus, it is also expected that network resilience is responsive to these environmental changes.

A large dataset of real-world ecological networks, constructed in [29], was used to evaluate the relationship between ecological network resilience and environmental changes. As in the study [6] on network resilience, the focus was on mutualistic networks (i.e. pollination networks and seed-dispersal networks), and the plant network resilience and animal network resilience of each plant–animal network were calculated. Spatial analysis was used to evaluate the contributions of environmental changes to ecological network resilience. Moreover, potential confounding effects were taken into consideration, in addition to the application of ordinary least-squares (OLS) regression analysis. As in [30], pollination networks and seed-dispersal networks were separately investigated because they differ in terms of animal species types. In particular, animals in pollination networks are mainly insects, whereas, those in seed-dispersal networks are mainly birds.

## 2. Methods

### 2.1. Mutualistic network dataset

A large dataset of real-world plant–animal mutualistic networks constructed in [29] was used. The dataset contained 62 pollination (plant–pollinator) networks and 30 seed-dispersal (plant–disperser) networks, which were collected from the supporting online material in [31], the Interaction Web DataBase (www.nceas.ucsb.edu/interactionweb/), and the Web-of-Life Database (www.web-of-life.es). The mutualistic networks were represented as bipartite networks because mutualistic links are drawn only between two types of organisms (i.e. plants and animals) [32]. These networks were represented as binary networks because the references included a large amount of binary data: approximately 71% (44/62) of pollination networks and approximately 53% (16/30) of seed-dispersal networks were binary.

### 2.2. Environmental data

In the dataset from [29], environmental data (i.e. climatic parameters, elevation, human impact and climate change velocities) were also available. The climatic parameters were obtained from the WorldClim database [33] (v. 1.4, release 3; www.worldclim.org) based on the latitudes and longitudes of identified observation sites at a spatial resolution of 2.5′. The values for each parameter were obtained from the coordinate centre. The available parameters were annual mean temperature (*T*_mean_) (×10°C), temperature seasonality (standard deviation) (*T*_seasonality_), annual precipitation (*P*_ann_) (mm), and precipitation, or rainfall seasonality (coefficient of variation) (*P*_seasonality_). Elevations (m) were extracted using the Google Elevation Application Programming Interface (developers.google.com/maps/documentation/elevation/). The human footprint (HF) score was used for evaluating human impact. HF scores are provided with a spatial resolution of 1 km grid cells in the ‘Last of the Wild Project’ [34] (version 2), and they were defined based on human population density, human land use and infrastructure (built-up areas, night-time lights, and land use or land cover), and human access (coastlines, roads, railroads and navigable rivers). Two types of historical climate-change velocities were considered: temperature-change velocity or warming velocity (*T*_velocity_), and precipitation-change velocity (*P*_velocity_). As in [28,35], climate-change velocity was defined as the temporal climate gradient divided by the spatial climate gradient, where the temporal gradient is defined as the absolute difference between the current and the CCSM3 model-based Last Glacial Maximum climate conditions, available in the WorldClim database (www.worldclim.org/past), and the spatial gradient was the local slope of the current climate surface at the study site, calculated using the R package *raster*.

### 2.3. Network resilience

As in [6], the network resilience of plant–animal mutualistic networks was calculated using the single macroscopic resilience parameter $\beta_{\mathrm{eff}}=\sum_{ij}A_{ij}A_{\;ji}/\sum_{ij}A_{ij}.$ The matrix *A_ij_* corresponds to the weighted plant (animal) network constructed by projecting the binary plant–animal bipartite network on the plant (animal) set. Plant network resilience (*β*_eff_ for plant networks) and animal network resilience (*β*_eff_ for animal networks) were calculated because the plant and animal networks were obtained from a plant–animal mutualistic network. If a plant–animal bipartite network consists of *n* plants and *m* animals, the matrix *A_ij_* for the *n* × *n* plant network (for the *m* × *m* animal network) is obtained as

$$A_{ij}=\overset{m}{\underset{k=1}{\sum }}\frac{M_{ik}M_{\;jk}}{\sum_{s=1}^{n}M_{sk}}\;\;\left(A_{ij}=\overset{n}{\underset{k=1}{\sum }}\frac{M_{ki}M_{kj}}{\sum_{s=1}^{m}M_{ks}}\right),$$

where *M_ik_* is the *n* × *m* incidence matrix of the bipartite mutualistic network. *M_ik_* = 1 if plant *i* interacts with animal *k* via mutualistic relationships, and *M_ik_* = 0 otherwise. That is, *A_ij_* for the plant (animal) network is the sum of the inverse degrees of common neighbours between plants (animals) *i* and *j* in the bipartite mutualistic network. According to [6], *A_ij_* indicates the weight of the interaction between *i* and *j*, and it is defined as the density of mutual symbiotic relationships between *i* and *j* based on the following concepts: (i) stronger mutualistic interaction between plants (animals) *i* and *j* are observed when the plants (animals) share more mutual animals (plants) *k*; (ii) by contrast, the contribution to each plant (animal) is smaller when animals (plants) *k* mutually interact with more plants (animals).

### 2.4. Statistical analysis

The statistical analysis was based on the procedures in [29]. To evaluate the contribution of each variable to network resilience (*β*_eff_), regression analysis was performed using the R software package, v. 3.4.3 (www.r-project.org). Both OLS regression and the spatial analysis approach were considered (electronic supplementary material, source code S1). For the OLS regression, full models were constructed encompassing all explanatory variables (*T*_mean,_ *T*_seasonality_, *P*_ann_, *P*_seasonality_, elevation, human impact (HF score), *T*_velocity_ and *P*_velocity_), and the best model was selected to obtain the most simplified (easy-to-interpret) model and simultaneously avoid multi-collinearity in the full model. The best model was selected based on the sample-size-corrected v. of the Akaike information criterion (AICc) values using the R package *MuMIn*, v. 1.15.6. To examine the effects of environmental factors on network resilience and statistically control the potentially confounding effects of network size, species richness, or the number of species *S*, was also considered according to [27–29]. As a single selected model is the best model, the importance of certain variables may be overestimated, whereas important variables may be overlooked. To avoid such a model selection bias, a model-averaging approach using *MuMIn* was adopted. The averaged model was obtained in the top 95% confidence set of models. A global Moran's test was performed to evaluate spatial autocorrelation in the regression residuals using the function *lm.morantest.exact* in the R package *spdep*, v. 0.6.13. As in [27,28], the following parameters were log-transformed: *S*, *T*_velocity_ and *P*_velocity_. *β*_eff_ was also log-transformed for normality. As in [30], *P*_ann_ was square-root transformed. The quantitative variables were normalized to the same scale, with a mean of 0 and standard deviation of 1, using the *scale* function in R before the analysis. When spatial correlation was concluded in the OLS model (the associated *p*-value of Moran's test was less than 0.05), a spatial eigenvector mapping (SEVM) modelling approach [36,37] was also considered to remove spatial autocorrelation in the regression residuals. Specifically, the Moran eigenvector approach was adopted using the function *SpatialFiltering* in the R package *spdep*. As with OLS regression analysis, full models were constructed, and then the best model was selected based on AICc values. The spatial filter was fixed in the model-selection procedures [36]. The averaged models were also obtained. The contribution (i.e. non-zero estimate) of each explanatory variable to network resilience was considered significant when the associated *p*-value was less than 0.05. The best and averaged models were used to evaluate the contribution of each variable to network resilience; however, the full model was also considered for comparison. The residuals of the explanatory variables and network resilience were generally calculated according to the SEVM modelling approach-based best models; however, they were obtained according to the OLS regression-based best model when animal network resilience was investigated in seed-dispersal networks.

## 3. Results

### 3.1. Pollination networks

Sixty-two pollination networks were investigated (electronic supplementary material, table S1). Spatial autocorrelation was concluded in the OLS regression analysis; thus, the SEVM modelling approach was adapted (tables 1 and 2).

**Table 1.** Influence of explanatory variables on plant network resilience in pollination networks. *T*_mean_ and *T*_seasonality_ indicate mean annual temperature and temperature seasonality, respectively; *P*_ann_ and *P*_seasonality_ represent annual precipitation and precipitation seasonality, respectively; *T*_velocity_ and *P*_velocity_ represent mean temperature-change (warming) velocity and precipitation-change velocity, respectively. The estimates in the full, best and averaged models based on the ordinary least squared (OLS) regression and spatial eigenvector mapping (SEVM) modelling approach are shown. *R*^2^ denotes the coefficient of determination for full and best models based on the OLS regression and SEVM modelling. Values in brackets are the associated *p*-values.

variables	OLS			SEVM		
	estimate (full)	estimate (best)	estimate (average)	estimate (full)	estimate (best)	estimate (average)
richness	0.383 (<0.01)	0.370 (<0.01)	0.370 (<0.01)	0.424 (<0.01)	0.373 (<0.01)	0.379 (<0.01)
elevation	−0.177 (0.31)	−0.187 (0.09)	−0.212 (0.13)	0.008 (0.96)		−0.077 (0.56)
*T*_mean_	−0.213 (0.34)		−0.137 (0.57)	−0.077 (0.71)		−0.028 (0.90)
*T*_seasonality_	0.189 (0.35)	0.371 (<0.01)	0.356 (0.01)	0.252 (0.21)	0.431 (<0.01)	0.405 (<0.01)
*P*_ann_	0.077 (0.58)		0.055 (0.66)	−0.085 (0.49)		−0.053 (0.65)
*P*_seasonality_	−0.031 (0.85)		−0.107 (0.46)	−0.143 (0.31)		−0.135 (0.24)
human impact	0.254 (0.07)	0.212 (0.05)	0.244 (0.05)	0.226 (0.08)	0.258 (0.01)	0.241 (0.02)
*T*_velocity_	0.040 (0.83)		0.152 (0.34)	0.071 (0.70)		0.146 (0.31)
*P*_velocity_	0.124 (0.34)		0.122 (0.33)	0.136 (0.26)		0.117 (0.30)
Moran's *I*	0.18 (0.01)	0.17 (0.02)		−0.22 (0.45)	−0.19 (0.57)	
*R*^2^	0.45 (<0.01)	0.43 (<0.01)		0.68 (<0.01)	0.66 (<0.01)

**Table 2.** Influence of explanatory variables on animal network resilience in pollination networks. See table 1 for description of table elements.

variables	OLS			SEVM		
	estimate (full)	estimate (best)	estimate (average)	estimate (full)	estimate (best)	estimate (average)
richness	0.576 (<0.01)	0.586 (<0.01)	0.574 (<0.01)	0.468 (<0.01)	0.512 (<0.01)	0.465 (<0.01)
elevation	0.268 (0.13)		0.208 (0.18)	0.206 (0.26)		0.127 (0.38)
*T*_mean_	0.542 (0.02)	0.548 (<0.01)	0.521 (0.01)	0.695 (<0.01)	0.536 (<0.01)	0.460 (0.01)
*T*_seasonality_	−0.130 (0.53)		−0.187 (0.40)	0.115 (0.55)		0.062 (0.72)
*P*_ann_	−0.421 (0.01)	−0.410 (<0.01)	−0.388 (0.01)	−0.234 (0.11)	−0.241 (0.06)	−0.218 (0.11)
*P*_seasonality_	−0.218 (0.20)		−0.149 (0.35)	0.034 (0.84)		0.092 (0.52)
human impact	−0.326 (0.02)	−0.324 (0.01)	−0.307 (0.03)	−0.214 (0.09)	−0.211 (0.05)	−0.191 (0.11)
*T*_velocity_	0.054 (0.78)		0.032 (0.87)	0.120 (0.51)		0.012 (0.99)
*P*_velocity_	0.136 (0.30)		0.103 (0.44)	0.032 (0.78)		0.056 (0.60)
Moran's *I*	0.18 (0.01)	0.24 (0.01)		−0.18 (0.47)	−0.15 (0.52)	
*R*^2^	0.43 (<0.01)	0.38 (<0.01)		0.60 (<0.01)	0.59 (<0.01)	

**Figure 1.**
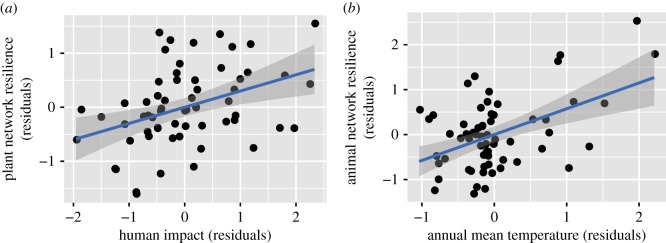
Scatter plots of network resilience (residuals) versus environmental parameters (residuals) in pollination networks. (*a*) Plant network resilience versus human impact. (*b*) Animal network resilience versus annual mean temperature.

The full, best and averaged models in spatial analysis indicated that both plant network resilience and animal (pollinator) network resilience increased with network size (species richness). More importantly, it was found that network resilience was associated with environmental factors. Specifically, the best and averaged models in the OLS regression analysis and spatial analysis demonstrated a positive correlation between plant network resilience and human impact (table 1 and figure 1*a*). In addition, plant network resilience was positively associated with temperature seasonality.

The full, best and averaged models in the OLS regression analysis and spatial analysis indicated that animal network resilience increased with mean annual temperature (table 2 and figure 1*b*). The best and averaged models in the OLS regression analysis indicated that animal network resilience was negatively associated with annual precipitation and human impact; however, spatial autocorrelation analysis suggested that the observed associations were in fact merely an artefact; no association between animal network resilience and annual precipitation or human impact was detected when spatial dependency was removed from the regression residuals (i.e. when an SEVM modelling approach was applied).

### 3.2. Seed-dispersal networks

Thirty seed-dispersal networks were investigated (electronic supplementary material, table S2).

**Figure 2.**
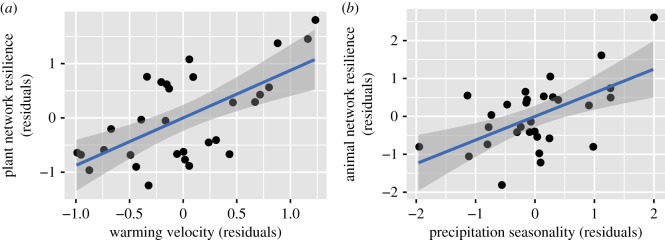
Scatter plots of plant network resilience (residuals) versus environmental parameters (residuals) in seed-dispersal networks. (*a*) Plant network resilience versus warming velocity. (*b*) Animal network resilience versus precipitation seasonality.

Spatial autocorrelation was concluded in the OLS regression analyses when the contribution of each variable to plant network resilience was evaluated; thus, spatial analysis was also performed (table 3). The full and best models in spatial analysis showed that a number of parameters were associated with plant network resilience. However, the averaged model indicated that plant network resilience was mainly affected by species richness, annual precipitation and warming velocity. The observed associations with elevation, annual mean temperature and precipitation seasonality were not statistically conserved in the top 95% confidence set of models. As in pollination networks, plant network resilience increased with species richness. More interestingly, plant network resilience increased in response to warming velocity (figure 2*a*); by contrast, it decreased with annual precipitation. The best model in the OLS regression analysis showed that plant network resilience was associated with temperature seasonality, precipitation seasonality and historical precipitation-change velocity; however, the averaged model in the OLS regression analysis and spatial autocorrelation analysis suggested that the observed associations were not statistically significant.

**Table 3.** Influence of explanatory variables on plant network resilience in seed-dispersal networks. See table 1 for description of table elements.

variables	OLS			SEVM		
	estimate (full)	estimate (best)	estimate (average)	estimate (full)	estimate (best)	estimate (average)
richness	0.430 (0.06)	0.399 (0.06)	0.286 (0.24)	0.585 (0.01)	0.563 (<0.01)	0.502 (0.03)
elevation	0.629 (0.10)		0.037 (0.92)	0.908 (0.01)	0.888 (<0.01)	0.727 (0.08)
*T*_mean_	0.922 (0.16)		0.392 (0.30)	1.302 (0.04)	1.188 (<0.01)	0.936 (0.05)
*T*_seasonality_	−0.099 (0.85)	−0.696 (0.01)	−0.491 (0.16)	0.110 (0.82)		−0.394 (0.40)
*P*_ann_	−0.562 (0.02)	−0.492 (0.04)	−0.390 (0.14)	−0.834 (<0.01)	−0.940 (<0.01)	−0.745 (0.01)
*P*_seasonality_	−0.695 (0.01)	−0.522 (0.02)	−0.402 (0.16)	−0.556 (0.02)	−0.332 (0.05)	−0.380 (0.13)
human impact	−0.136 (0.45)		−0.265 (0.17)	−0.068 (0.67)		−0.204 (0.31)
*T*_velocity_	0.482 (0.09)		0.211 (0.37)	0.809 (0.01)	0.875 (<0.01)	0.665 (0.04)
*P*_velocity_	0.563 (0.04)	0.711 (<0.01)	0.475 (0.10)	0.291 (0.27)		0.326 (0.22)
Moran's *I*	0.17 (0.04)	0.26 (0.02)		−0.19 (0.49)	−0.14 (0.48)	
*R*^2^	0.53 (0.04)	0.43 (0.01)		0.65 (0.01)	0.61 (<0.01)	

For animal (disperser) network resilience, only the OLS regression approach was considered because spatial autocorrelation was not concluded (table 4). The full and best models indicated that animal network resilience increased with precipitation seasonality (figure 2*b*) and decreased with historical precipitation-change velocity. However, the averaged model suggested that the observed association between animal network resilience and historical precipitation-change velocity was not statistically significant; rather, it indicated that precipitation seasonality mainly affected animal network resilience.

**Table 4.** Influence of explanatory variables on animal network resilience in seed-dispersal networks. See table 1 for description of table elements.

variables	OLS		
	estimate (full)	estimate (best)	estimate (average)
richness	0.384 (0.05)	0.294 (0.07)	0.267 (0.19)
elevation	−0.605 (0.07)		−0.329 (0.23)
*T*_mean_	0.122 (0.83)		0.419 (0.32)
*T*_seasonality_	0.703 (0.17)		0.266 (0.64)
*P*_ann_	0.297 (0.14)		0.296 (0.17)
*P*_seasonality_	0.744 (<0.01)	0.617 (<0.01)	0.506 (<0.05)
human impact	−0.207 (0.20)		−0.189 (0.30)
*T*_velocity_	−0.402 (0.12)		−0.271 (0.28)
*P*_velocity_	−0.713 (0.01)	−0.534 (0.01)	−0.485 (0.07)
Moran's *I*	−0.38 (0.97)	−0.09 (0.57)	
*R*^2^	0.61 (0.01)	0.38 (0.01)	

## 4. Discussion

As suggested in [6], the network resilience of mutualistic ecosystems was empirically investigated. It was confirmed that network resilience increased with network size (species richness). This is consistent with a number of previous studies [38,39]. Moreover, it was hypothesized that the network resilience of mutualistic ecosystems is associated with environmental changes such as climate change velocity and human impact, and this hypothesis was tested. As expected, it was found that network resilience was associated with warming velocity and human impact. In particular, the plant network resilience of pollination networks and seed-dispersal networks increased with human impact and warming velocity, respectively. However, animal network resilience was associated with current climate rather than warming velocity and human impact. This may be due to the fact that mutualistic networks are more sensitive to plant than to animal extinction under climate change. Schleuning *et al*. [18] demonstrated that projected plant extinctions (under climate change) are more likely to trigger animal coextinctions than vice versa. This result indicated that the impact of climate change on biodiversity was amplified via extinction cascades from plants to animals in mutualistic networks. The focus in [18] was on current human-driven climate change; thus, this study may mainly support the finding that human impact on network resilience was remarkable for plants. However, it may also be applicable to historical climate change (i.e. warming velocity) because the impact amplified via extinction cascades from plants to animals may be general. That is, plant network resilience, rather than animal network resilience, should increase so that mutualistic networks may remain stable despite environmental changes.

These results indicate that real-world mutualistic networks have a structure that increases ecosystem resilience against environmental changes. This is related to the optimal principles of ecological networks, as several theoretical studies reported. For example, real-world ecological network structure may minimize competition and increase biodiversity [23], and emerge as a result of an optimization principle aimed at maximizing species abundance [25], despite some criticism in [40–42].

Alternative hypotheses should also be considered, particularly in regard to the relationship between current climate and mutualistic networks. Specifically, climate seasonality also affected mutualistic network resilience, and network resilience increased with temperature seasonality in pollination networks. Moreover, animal network resilience was positively associated with precipitation seasonality in seed-dispersal networks. Given that climate seasonality can also be considered an environmental perturbation [26], it is predicted that mutualistic networks are generally adapted to changing environmental conditions. The observed associations suggest that real-world mutualistic networks also have a structure that increases ecosystem resilience against climate seasonality, consistent with such a prediction.

Annual climatic parameters also affected network resilience in mutualistic ecosystems. Plant network resilience decreased with annual precipitation in seed-dispersal networks. This may be due to the fact that the interactions between plants were weakened owing to rainfall. According to the definition of interaction strength (i.e. link weight) for mutualistic networks in [6] (see also §2.3), the interactions in plant networks were stronger when the plants shared more mutual animals. Animals may find it difficult to visit plants during rains. In this case, link weight decreases; as a result, the networks are less resilient. In pollination networks, animal network resilience increased with mean annual temperature. This may also be due to the change in the interaction strength owing to the difference in climate conditions. By [6], the link weight (interaction strength) in animal networks is defined based on the number of shared mutual plants. At warmer sites, plant abundance may be higher because animals (pollinators) may more actively visit plants [43]; as a result, animals may share more mutual plants. In such a case, link weight increases, and thus the networks are more resilient. Several studies [28–30] reported that annual precipitation and annual mean temperatures also altered mutualistic network structure.

However, more careful examinations may be required to understand the relationship between mutualistic network resilience and environmental factors. For example, the definition of network resilience (*β*_eff_) is still controversial. In particular, two main conditions are assumed, namely, the network determined by the interaction between pairs of nodes (species) has negligible degree correlations, where degree indicates the number of links per species, and the node activities are uniform across nodes on both the drift and the pairwise interaction functions (i.e. the self-dynamics and interaction dynamics should be considered linear in their variables). These conditions may pose problems because the variability of the conditions has already been evaluated using real-world mutualistic network data. Tu *et al*. [44] demonstrated the limited effects of the conditions on the errors of the approximation framework; however, they also showed that the conditions are neither sufficient nor necessary to ensure that their method is applicable in general, and the validity of their results is not independent of the multi-dimensional system of equations that Gao *et al*. [6] considered. The validity of network resilience (i.e. the approach for evaluating ecosystem reliance using ecological networks) is still debatable in a theoretical context. Further development of the theory is awaited to evaluate ecological network resilience under more realistic conditions.

The time-scale of the climate change velocities may be overly long in terms of ecological-network assemblages because the velocities were estimated based on the difference between the current and last glacial maximum climate conditions according to [27,28,45]. As mentioned previously [27,29], this may be due to the fact that one of the strongest climatic shifts has occurred since the last glacial maximum (21 000 BP). The climatic shift has influenced geographical patterns of species endemism [35], suggesting that species composition (and hence ecological-network assemblages) are more susceptible to environmental perturbations in areas that have experienced larger climatic shifts. However, it is also important to consider short-range climate-change velocity. For example, the velocity of temperature change [46], derived from spatial gradients and multi-model ensemble forecasts of the rate of temperature increase over the twenty-first century, may be useful; however, the short-range velocity was not examined owing to the data unavailability.

As mentioned in [26,29], the present analysis has several limitations, as many other analyses of ecological networks. For example, the interaction strength or weights in mutualistic (bipartite) networks were not considered, although it is also important to consider a weighted network analysis, as a different conclusion may be derived from comparisons between weighted networks and binary networks [41,45]. This is due to the fact that the datasets that were used included a large amount of binary data, and the amount of data on weighted networks was insufficient for spatial analysis. Moreover, the definition of interaction weight is not uniform throughout the ecological-network datasets. Therefore, a binary network approach was adopted to represent all ecological networks, so that issues resulting from these variations might be avoided. As in [6], the focus was only on mutualistic ecosystems; thus, the mixture of interaction types (i.e. antagonistic interactions and mutualistic interactions) was ignored, although it is more representative in real-world ecosystems [2,47], and the multi-layer nature of ecological networks [48] has recently been intensively investigated. Network resilience for multiple network types should be considered in the future to evaluate ecological network resilience under more realistic conditions. The sampling effort may affect network resilience owing to the species–area relationship [49], which states that the number of observed species increases with an increase in the observed area. When the dataset in [29] was constructed, the relevant information on sampling effort could not be obtained because the data were not always clearly delineated in the literature. However, this limitation poses little problem because the effect of the number of species was removed from the statistical analysis, and [30] suggested that network parameters are mostly independent of sampling effort (observation area and observation time). In addition, the effects of phylogenetic signals were not considered because species descriptions in the networks are partially unknown or ambiguous. However, the absence of phylogenetic signals is unlikely to have a significant effect, as several studies have reported that phylogenetic signals are weak in ecological networks [45,50]. Moreover, a restricted understanding of interspecific reactions is a more serious limitation. To avoid these limitations, larger-scale and more highly normalized databases should be constructed. In this context, data sharing [51] may be important.

Despite the limitations of the theory and data analysis, these findings enhance the understanding of the structure and resilience of ecosystems. Furthermore, they indicate the possible application of the theory for biodiversity maintenance and environmental assessment; in particular, the macroscopic resilience parameter (i.e. network resilience) *β*_eff_ may be a useful index in evaluating ecosystem resilience against environmental change.

## Supplementary Material

Table S1. List of pollination networks.

## Supplementary Material

Table S2. List of seed-dispersal networks.

## Supplementary Material

Source code S1. An example R script for data analysis.

## Data accessibility

The datasets supporting this article have been uploaded as electronic supplementary material.
